# Neuroprotective Effect of Intrastriatal Caffeic Acid Phenethyl Ester Treatment in 6-OH Dopamine Model of Parkinson's Disease in Rats

**DOI:** 10.1155/2021/5553480

**Published:** 2021-08-30

**Authors:** Burak Cem Soner, Eda Acikgoz, Salim Yalcin Inan, Sule Ayla, Ayse Saide Sahin, Gulperi Oktem

**Affiliations:** ^1^Izmir Democracy University, Faculty of Medicine, Department of Pharmacology, 35100 Izmir, Turkey; ^2^Van Yuzuncu Yil University, Faculty of Medicine, Department of Histology and Embryology, 65080 Van, Turkey; ^3^Necmettin Erbakan University, Meram Faculty of Medicine, Department of Medical Pharmacology, 42100 Konya, Turkey; ^4^Istanbul Medipol University, School of Medicine, Department of Histology and Embryology, İstanbul, Turkey; ^5^Ege University, Faculty of Medicine, Department of Histology and Embryology, 35100 Izmir, Turkey

## Abstract

Parkinson's disease (PD) is the second most common neurodegenerative disorder, and the main cause of PD is still not known. Until now, no cure for Parkinson's disease is yet in sight. Caffeic acid phenethyl ester (CAPE) is a polyphenolic component of the propolis, which can be derived from honeybee hive propolis. We aimed to determine the effect of intrastriatal CAPE administration as a neuroprotective agent on 6-hydroxydopamine (6-OHDA)-induced PD model. Adult male Wistar rats weighing 280–320 g were used. The PD model was induced with unilateral intrastriatal 6-OHDA injection. Treatment groups received 20 *μ*mol/5 *μ*L/4 day and 80 *μ*mol/5 *μ*L/4 day CAPE 24 h after 6-OHDA injection. Eight days after 6-OHDA application, behavioral studies (adhesive tape removal test, open-field test, cylinder test, and apomorphine-induced asymmetric rotational behavior) were performed once more to compare the effects of CAPE on behavior tests. Striatal histological verifications, immunohistochemistry, and stereological quantitation were performed. Our results for the first time showed that, besides improving the motor performance, CAPE treatment also prevents 6-OHDA-induced loss of TH-positive neurons. From our results, CAPE may be a promising clinical agent in the treatment of PD.

## 1. Introduction

Parkinson's disease (PD) is defined as the second most common neurodegenerative disorder after Alzheimer's disease and is characterized by the progressive loss of dopaminergic neurons in substantia nigra pars compacta. The main cause of PD is still not known. Oxidative stress seems to be the most prevalent mechanism, but growing evidence shows that oxidative stress-targeted activation of mitochondrion-dependent programmed cell death and apoptosis seems to be responsible for the disease [1–3]. Clinical signs of the disease appear after a 60–65% dopaminergic neuronal loss at substantia nigra, and therefore a 80–85% dopamine level decrease at striatum [4]. The progression of the disease may vary from patient to patient. Studies about the rate of the disease show that the median time to end-stage PD from the onset of symptoms is 9 to 14 years. 50% slowing down in the progression of the disease will have a 35% decrease in the economic implications and minimize the negative impact of the symptoms [5]. Until now, no cure for Parkinson's disease is yet in sight. Treatment modalities are mostly based on reducing the symptoms and/or stimulation of neurodegenerated dopaminergic neurons, [6] and, therefore, current research strategies and treatment approaches are headed towards the prevention of dopaminergic neuronal loss [7].

Caffeic acid phenethyl ester (CAPE) is a polyphenolic component of the propolis, which can be derived from honeybee hive propolis. Antioxidant, immunomodulatory, antiviral, and anti-inflammatory properties of CAPE have already been shown [8]. In recent years, the neuroprotective potential of CAPE has also been studied. In vitro protective effect of 10 *μ*M CAPE on rat mesencephalic neurons and cerebellar granule neurons has been shown [9, 10]. Some studies have attributed this neuroprotective potential to its effect on inflammation [11], mitochondrial cytochrome c release [8, 9], blocking free radical generation [10, 12], autoimmune processes [13], and protection of mitochondrial dysfunction and reduction of caspase-9 activity [12].

In this study, we aimed to determine the effect of intrastriatal CAPE administration as a neuroprotective agent on 6-hydroxydopamine (6-OHDA)-induced PD rat model and also to evaluate the effects of CAPE treatment on behavior tests and histological verification.

## 2. Material and Methods

### 2.1. Chemicals

CAPE (C8221) and 6-OHDA (H116; contains 0.01% (w/v) ascorbic acid as stabilizer) were purchased from Sigma-Aldrich Corporation, St. Louis, MO, USA. CAPE was dissolved in ethanol and further dilutions were made in saline. Chemicals used for histological verification were purchased from Sigma (Germany). TH was obtained from Bioss, USA (bs-0016R).

### 2.2. Animals and Experiment Schedule

**Figure 1:**
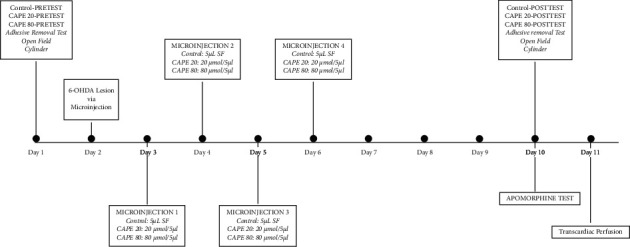
The experimental schedule.

Adult male Wistar rats weighing 280–320 g were obtained from the Animal Care facility of Meram Faculty of Medicine. Rats were housed individually in standard translucent plastic cages and kept in an environmentally controlled vivarium under a 12 : 12-h light-dark cycle. They were given food and water ad libitum. Experiments were conducted between 09 : 00 and 16 : 00 h. Rats were allowed for a 1 h adaptation period for the laboratory conditions before behavioral testing, and they were randomly assigned to three groups as: group1: 6-OHDA lesion + 5 *μ*L/4 day saline treated PD group (control group, *n* = 7), group 2: 6-OHDA lesion + 20 *μ*mol/5 *μ*L/4 day CAPE treated group (CAPE 20 group, *n* = 9); and group 3: 6-OHDA + 80 *μ*mol/5 *μ*L/4 day CAPE treatment group (CAPE 80 group, *n* = 8). The individual behavior tests were performed as follows: basal values were evaluated at day 1, and the effect of the CAPE treatment was evaluated at day 10. All procedures conformed to the National Institute of Health Guide for the Care and Use of Laboratory Animals (NIH Publications No. 80–23) guidelines and were approved by local Animal Care and Use Committee (Protocol 2013–146). The experimental schedule is given in Figure 1.

### 2.3. Stereotaxic Surgery

Stereotaxic surgery was performed by the following standard operation procedures [14]. Briefly, rats were anesthetized with an intramuscular ketamine (80 mg/kg) + xylazine (10 mg/kg) mixture and secured in a stereotaxic frame (Stoelting, USA). The skull was leveled between bregma and lambda. The level of anesthesia was monitored by testing corneal reflexes and motor responses to tail pinch. Supplemental injections were administered as required. A stainless-steel guide cannula (11 mm, 21 G) was unilaterally implanted to the right hemisphere at the following coordinates: AP: −1.80 mm, ML: +2.10 mm from bregma, and DV: −0.60 mm below skull [15]. The guide cannula has been fixed with dental acrylic to two stainless steel studs screwed into the skull of the rats. A removable dummy cannula (27 G) was inserted to prevent occlusions. At the end of the surgery, local antibiotic (bacitracin) and saline (0.5 ml) + 5% sucrose (0.5 ml) were administered to rats.

### 2.4. Unilateral Intrastriatal 6-OHDA and CAPE Microinjections

After stereotaxic surgery, all animals were lesioned by 8 *μ*g/4 *μ*l 6-OHDA injection via a guide cannula (AP: −1.80 mm, ML: +2.10 mm from bregma, and DV: −7.70 mm) with a duration of 1 *μ*l/min [16]. At the end of the infusion, the needle was kept in place 2 min more to let the toxin diffuse from the lesion site and, then, was slowly retracted.

24 hours after 6-OHDA injection, each rat received 4 microinjections from day 2 to day 5 (Figure 1.). The control group received saline, CAPE 20 group received 20 *μ*mol/5 *μ*L/day CAPE, and CAPE 80 group received 80 *μ*mol/5 *μ*L/day CAPE.

Each microinjection was carefully performed, and CAPE or saline were simultaneously infused into right hemisphere in a final volume of 5 *μ*l over 5 min. The infusion cannula was cut from 27G Hamilton needles mounted to 10 *μ*l Hamilton injectors (Hamilton Company, USA). After microinjections, the needle was kept in place 2 min more to allow the diffusion of the drug.

### 2.5. Behavioral Studies

#### 2.5.1. Adhesive Tape Removal Test

An adhesive tape removal test is used to evaluate the sensorimotor integration and has been adapted from the method proposed by Sughrue et al. [17] In brief, two self-adhesive tapes of equal size are affixed on the distal region of the paws to stimulate bilateral tactile stimuli. For a 15 min period, the total time that the animal spends attempting to remove the tapes for each paw were recorded. Baseline data (termed as pretest) were evaluated at day 1. The test was reperformed at day 10. Time spent attending to this stimulus is counted. The ratio of the contralateral side of the lesion (left side) time versus total time were calculated to quantify the damage. The scoring was performed by a blind evaluator.

#### 2.5.2. Open-Field Test

Spontaneous locomotor activity was investigated in the open-field as previously described [18]. Briefly, the open-field apparatus was a white square arena (90 cm × 90 cm × 40 cm) divided into 25 small equal units. Rats were gently placed individually into the open-field facing one corner and allowed to explore the area for 5 min. No baseline trials were required for this test. The activity level was expressed as the total number of squares crossed during a 5 min testing period [19]. The test was performed at day 1 and at day 10. The scoring was performed by a blind evaluator.

#### 2.5.3. Cylinder Test

The cylinder test, namely, the forelimb asymmetry test, was conducted using the protocol from that described previously [20, 21]. Briefly, 5 min after the open-field test, rats were gently placed individually into the clear plexiglass cylinder (diameter 21 cm × height 30 cm) for 5 min, and following behavioral parameters were video-recorded for further analyses. The number of times each forepaw comes in contact with the cylinder walls is counted. Data were presented as left forepaw contacts as the percentage of the total number of contacts of each forepaw. No baseline trials were required for this test. The test was performed at day 1 and at day 10. The scoring was performed by a blind evaluator.

#### 2.5.4. Apomorphine-Induced Asymmetric Rotational Behavior

Drug-induced rotational behavioral effect (turning behavior) for nigrostriatal neurodegeneration has been evaluated after 8 days of lesioning (day 10). 0.25 mg/kg apomorphine has been given subcutaneously, and contralateral rotations of animals were recorded. The number of complete turns performed by the animals were counted for 40 min intervals. The results were expressed in rotations/40 min [22].

### 2.6. Histological Verification

Twenty-four hours after apomorphine-induced asymmetric rotational behavior testing, rats were deeply anesthetized with phenobarbital and transcardially perfused with 100 ml 0.1 M phosphate buffer saline (pH: 7.2–7.4) followed by 250 ml 4% paraformaldehyde (pH: 7.2–7.4). Brains were removed, postfixed in 4% paraformaldehyde overnight, then cryoprotected in 30% sucrose for 4 days. Coronal brain sections were cut in 5 *μ*m thickness on a cryostat (Leica Biosystems, Germany) and stored in 0.1 M phosphate buffer saline containing 0.02% sodium azide. Sections were mounted on gelatine-coated slides, air dried, dehydrated, and defatted in serial alcohol concentrations (50, 70, 95, and 100%) and xylene, respectively. Sections were then stained with cresyl violet, dehydrated and cover slipped with DPX permount for histological verification of the microinjections according to the rat brain atlas [15].

### 2.7. Immunohistochemistry and Stereological Quantitation

Brain samples were fixed in 4% paraformaldehyde, embedded with paraffin, sliced into 5 *μ*m section. Immunohistochemistry studies were performed by the following procedure: samples were incubated for 3 hours at 58°C. The samples were placed in hot xylol overnight at room temperature. In the morning, samples were placed in a microwave at 360°W for 2 min, and then the sample was cooled. The samples were washed with 80%, 95%, 100% distilled water and 1x PBS. The samples were placed in citrate buffer (pH 6.0; tween 20) and microwaved at 90°W for 5 min and 360°W for 15 min. Then, the samples were cooled and washed with 1x PBS followed by the addition of 3% (v/v) H_2_O_2_ in methanol at room temperature for 10 min and were washed with 1x PBS and incubated in blocking serum for 30 min. Then, the primary antibody [tyrosine hydroxylase (TH)] was added and incubated overnight at +4°C ( the indoor environment was kept moist). The next day, the secondary antibody was added for 30 min, and then the samples were washed with PBS (4–5 times) followed by streptavidin complex for 30 min. Then, after washing again with PBS, the DAP substrate chromogen solution was applied for 5–10 min. Finally, the slides were washed, and counterstained with Mayer's hematoxylin for 2 min and washed thoroughly with running tap water and distilled water. The slides were dehydrated (80%, 95%, 100% alcohol) and then placed in xylol for maximum 30 min, and the mounting medium was applied.

Stereological quantitation is performed after all sections were washed with PBS. Later, sections were photographed using an Olympus C-5050 digital camera mounted on an Olympus BX51 microscope. Immunoreactivity in the striatal sections was quantified by measuring the optical density (OD) per area using Image J software (National Institutes of Health, Bethesda, MD) as described before [23].

### 2.8. Statistical Analysis

Results were presented as means ± S.E.M. Statistical analyses were performed using GraphPad Prism (Graph Pad Software, La Jolla, CA, USA). The one-way analysis of variance followed by a posthoc Tukey test was used for comparing multiple groups, and the Student's *t*-test was used for comparing two groups. The statistical significance was accepted as *p* < 0.05 and *p* < 0.01.

## 3. Results

### 3.1. Adhesive Tape Removal Test

**Figure 2:**
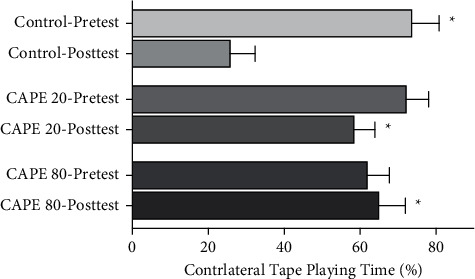
The ratio of contralateral lesion side (left side) tape playing time versus total playing time. Adhesive tape removal test results of control, CAPE 20, and CAPE 80 groups before (termed as pretest) and after (termed as posttest) 6-OHDA application. Control group, *n* = 7; CAPE 20 group, *n* = 9; CAPE 80 group, *n* = 8. ^*∗*^*p* < 0.01 versus control posttest.

The adhesive tape removal test is performed to investigate the movement asymmetries resulting from unilateral nigrostriatal damage. During our pretests (before 6-OHDA application), the ratios of contralateral/ipsilateral performances for control, CAPE 20, and CAPE 80 groups were similar (74.0 ± 6.8%, 72.6 ± 5.5%, and 62.3 ± 5.4%, respectively). Eight days after 6-OHDA application, the contralateral paw use showed a significant decrease in the control group (6-OHDA-induced lesion group), when compared with its pretest results (*p* < 0.01). Posttest results of the CAPE treated groups showed no difference when compared with their pretest results. There was a significant difference in the posttest results of the control group versus both CAPE groups (*p* < 0.01, control posttest versus CAPE 20-posttest and CAPE 80-posttest). Posttest results for control, CAPE 20, and CAPE 80 groups were 26.2 ± 6.1%, 58.8 ± 5.1%, and 65.4 ± 6.5%, respectively (Figure 2).

### 3.2. Open-Field Test

**Figure 3:**
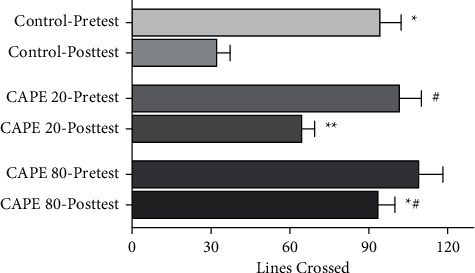
The numbers show the activity level as the total number of squares crossed during a 5 min testing period. Intrastriatal CAPE treatment (20 and 80 *μ*mol/5 *μ*L) reversed the decreases in locomotor activity observed in the 6-OHDA-lesioned group, and this protective effect was higher in the CAPE 80 group. Control group, *n* = 7; CAPE 20 group, *n* = 9; CAPE 80 group, *n* = 8 (^*∗*^*p* < 0.01 versus control posttest, ^*∗∗*^*p* < 0.05 versus control posttest; ^#^*p* < 0.05 versus CAPE 20 posttest).

The results indicated the neuroprotective effect of CAPE on 6-OHDA-induced nigrostriatal damage in the locomotor parameter. Eight days after 6-OHDA-lesion, the control group showed a decline in the locomotor activity (32.9 ± 4.5) when compared with its pretest results (94.9 ± 7.5) (*p* < 0.01). CAPE 20 group also showed a slight decrease (65.1 ± 4.3) when compared with its pretest results (102.3 ± 7.7), but the results were significantly higher than the control-posttest results (*p* < 0.05, CAPE 20-posttest vs control-posttest and CAPE 20-pretest). 80 *μ*mol/5 *μ*L CAPE treatment showed a full neuroprotection (94.1 ± 5.8) when compared with CAPE 20-posttest (*p* < 0.05) and control-posttest (*p* < 0.01) (Figure 3).

### 3.3. Cylinder Test

**Figure 4:**
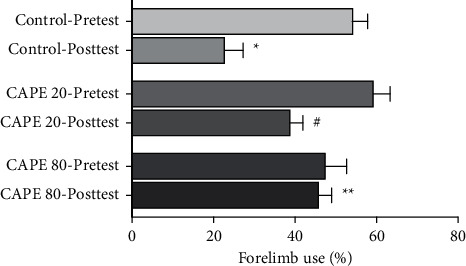
Data were presented as the left forepaw contacts as a percentage of the total number of contacts of each forepaw. Preferences in the forelimb usage of groups are given. Control group, *n* = 7; CAPE 20 group, *n* = 9; CAPE 80 group, *n* = 8 (^*∗*^*p* < 0.01 versus control-pretest; ^#^*p* < 0.05 versus CAPE 20-pretest; and ^*∗∗*^*p* < 0.01 versus control posttest).

The cylinder test was performed to evaluate the preference in forelimb usage. In our experiment, the impaired limb was the left forelimb (contralateral to the lesion). Pretest ratios for left forelimb usage showed no significant differences between control, CAPE 20, and CAPE 80 groups (54.5 ± 3.3%, 59.6 ± 3.7%, and 47.8 ± 4.8%, respectively; *p* > 0.05). 8 days after 6-OHDA administration, the control (6-OHDA-induced lesion rats) group showed a significant reduction in the independent use of the compromised (left, contralateral) forelimb in the cylinder (23.1 ± 4.2%, *p* < 0.01 versus control-pretest). This impaired contralateral forelimb use was recovered by 80 *μ*mol/5 *μ*L/4 days intrastriatal CAPE treatment (46.1 ± 2.8%, *p* < 0.01 versus CAPE 80-pretest). Although 20 *μ*mol/5 *μ*L intrastriatal CAPE treatment showed a modest increase with the impaired forelimb use, this increase was not significant (39.1 ± 2.8%) (Figure 4).

### 3.4. Apomorphine-Induced Asymmetric Rotational Behavior

**Figure 5:**
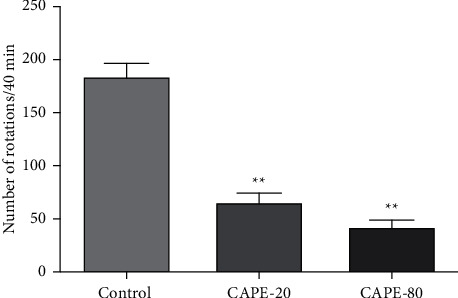
0.25 mg/kg apomorphine-induced contralateral rotations in 40 min for control (6-OHDA-induced lesion rats), CAPE 20, and CAPE 80 groups. Control group, *n* = 7; CAPE 20 group, *n* = 9; CAPE 80 group, *n* = 8. ^*∗∗*^*p* < 0.01 versus control.

At the 10^th^ day, we measured the contralateral rotation response to apomorphine to determine the extent of lesioning in rats. Subcutaneous 0.25 mg/kg apomorphine was given to rats. The number of contralateral rotations for 40 min were 184.4 ± 12.4, 66.1 ± 8.7, and 42.5 ± 6.7 for control, CAPE 20, and CAPE 80 groups, respectively. The control group showed a significantly higher number of rotations versus both treatment groups (*p* < 0.01) (Figure 5). No significant difference was found in rotations of CAPE 20 and CAPE 80 treated groups. These data suggest that the intrastriatal CAPE treatment protects striatal dopaminergic neurons both at 20 *μ*mol/5 *μ*L and 80 *μ*mol/5 *μ*L doses.

### 3.5. Histological Analysis of Striatal Neuron Numbers

**Figure 6:**
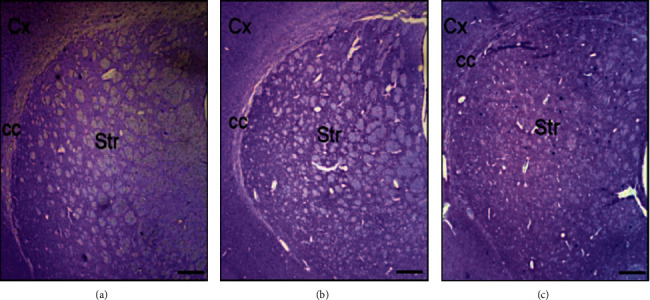
(a) Control group (6-OHDA-induced lesion rats); (b) CAPE 20 group; (c) CAPE 80 group. cc: corpus callusum, Cx : cortex, Str: striatum. Magnification ×400. Internal scale bar = 500 *μ*m.

Histological studies showed that (Table 1 and Figure 6) both 20 *μ*mol/5 *μ*L and 80 *μ*mol/5 *μ*L doses of intrastriatal CAPE treatment protect neurons 8 days after 6-OHDA injection in striatum when compared with the control group (*p* < 0.05 versus CAPE 20 group; *p* < 0.01 versus CAPE 80 group). The number of neurons in the CAPE 20 group also showed an attenuation when compared with the CAPE 80 group, showing that the therapeutical effect of CAPE is dose dependent. The number of neurons counted was 1241.7 ± 62.4, 1402.5 ± 31.4, and 1796.7 ± 75.1 for control, CAPE 20, and CAPE 80 groups, respectively.

### 3.6. Tyrosine Hydroxylase Immunoreactivity in Striatum

**Figure 7:**
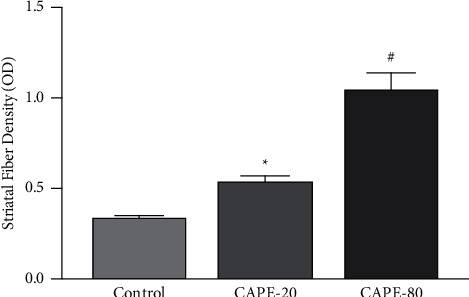
TH-immunostained striatal fiber density (OD) quantified using ImageJ. Control group, *n* = 7; CAPE 20 group, *n* = 9; CAPE 80 group, *n* = 8. ^*∗*^*p* < 0.05 CAPE-20 vs. control; and ^#^*p* < 0.01 vs. CAPE-80 vs control.

Intrastriatal TH immunoreactivity (TH-IR) was determined by immunohistochemical staining. Optic density of TH was 0.346 ± 0.012, 0.543 ± 0.079, and 1.054 ± 0.241 for control, CAPE 20, and CAPE 80 groups, respectively. Our results showed that eight days after intrastriatal 6-OHDA injection, TH-IR has decreased significantly in the control group in comparison to the CAPE 80 groups (*p* < 0.05) (Figure 7). Although 20 *μ*mol/5 *μ*L intrastriatal CAPE treatments showed a modest increase in TH-IR, this increase was not significant. From our results, we showed that intrastriatal CAPE treatment resulted with a dose dependent protection in TH immunoreactivity of dopaminergic striatal fiber density (Figure 7).

## 4. Discussion

Our results showed that, besides improving the motor performance, CAPE treatment also inhibits 6-OHDA-induced neurotoxicity. CAPE treatment prevents 6-OHDA-induced loss of TH-positive neurons. These results for the first time established the intrastriatal protective dose of CAPE and its in vivo protecting effect on behavioral tests against 6-OHDA mediated PD model in rats.

Unilateral injection of 6-OHDA results with an anterograde striatal degeneration, which proceeds within 12 h after injection and significant striatal terminal loss occurs after 2–3 days [24, 25]. Tyrosine hydroxylase is the rate limiting enzyme in catecholamine biosynthesis and also a marker for dopamine neurons. The number of TH-positive neurons and decrease in the immunoreactivity is the marker of 6-OHDA-induced PD [26]. Most studies showed apomorphine-induced contralateral rotation to evaluate the rating of striatal lesion and success of the treatment [27, 28]. This contralateral turning behavior is because of the supersensitivity of postsynaptic dopamine receptors (D1 and D2) on the lesioned side. We demonstrated that CAPE alleviates apomorphine-induced asymmetric rotational behavior, thus suggesting its protective effect on 6-OHDA-induced damage of striatal neurons in the rat Parkinson model.

Rats with a unilateral PD model avoid using their contralateral paw, and the cylinder test is used to highlight this defect by the asymmetries in front paw use. An adhesive tape removal test is first designed to evaluate the unilateral nigrostriatal damage [29], but it has been adopted for various diseases such as brain trauma, spinal cord lesion, and stroke. From our results, our modified adhesive tape removal test is a useful method to assess sensory and motor deficits in the 6-OHDA-induced unilateral PD model in rats. In rodent models, an open-field test is commonly used to evaluate locomotor behavior and is expected to deteriorate in several diseases like Parkinson's and Huntington's diseases. Our results have shown an increasing locomotor activity and an improvement in motor performance in open-field test, suggesting the neuroprotective effect of CAPE in the 6-OHDA-induced PD model.

6-OHDA is taken by dopamine transporters, which results in H_2_O_2_ and p-quinone derivative formation. It has also been shown that 6-OHDA is a mitochondrial complex I inhibitor. Because of its similarity with Parkinson's disease-like cell loss, 6-OHDA is frequently used to model PD. The 6-OHDA-induced PD model is accepted as an essential model to evaluate the new anti-Parkinsonian drugs [30] and new promising therapies [31–33].

In previous studies, the protective effects of CAPE have been evaluated in different application routes. Oral administrations via gastric gavage, or as a supplementation with food [34] or drinking water [35], or i.p administrations have been studied [36]. It is known that many drug candidates with high in vitro neuroprotective activity cannot be used as drugs, since they are unable to reach CNS at proper concentrations. Previous studies on the neuroprotective effect of CAPE after oral and parenteral applications expresses that CAPE crosses the blood brain barrier (BBB). The study of Silva et al. also support this with their model of the unilateral intrastriatal 6-OHDA PD model and its treatment via i.p. CAPE treatment [37]. Wang et al. studied the pharmacokinetics of CAPE in rat models. In their study, they have administered i.v 5 mg/kg to 20 mg/kg CAPE. Even the elimination half-life did not change in a dose-dependent manner, the volume of distribution changed and decreased significantly by about 70% as CAPE dose increased [38]. Another remarkable point is the rapid hydrolyses of CAPE to caffeic acid in rat plasma by carboxylesterase, which does not happen in human plasma [39, 40]. CAPE and caffeic acid both have antioxidant effects [41], and as both are lipophilic small phenolic compounds, they are expected to show a similar CNS penetration. From our point of view, this pharmacokinetic feature of CAPE in the rat model shown by Wang et al. stated that the dose applied and the amount of CAPE reaching the target tissue may differ in studies on the active substance. Detailed pharmacokinetic studies may be needed to clarify the concentration/efficacy of CAPE on CNS.

According to our research, until now, there is no information about central tissue concentrations of CAPE after peroral or parenteral administration. Our study pointed out that intrastriatal CAPE treatment can be achieved with a 80 *μ*mol concentration, independent of systemic administration, which may show variances depending on the dose.

Although protective effect's pathway and mechanism, which has not been demonstrated in our study, may be a limitation, our results for the first time showed that 20 *μ*mol/5 *μ*L and 80 *μ*mol/5 mL intrastriatal CAPE treatment doses, noticeably the 80 *μ*mol/5 mL dose, dependently showed a clear locomotor improvement and protected neuronal injury in 6-OHDA rat model of PD.

## Figures and Tables

**Table 1:** Number of neurons 8 days after 6-OHDA injection for control, CAPE 20, and CAPE 80 groups. ^*∗*^*p* < 0.05, ^#^*p* < 0.001 (versus control), ^†^*p* < 0.001 (versus CAPE 20 group).

	Control group	CAPE 20 group	CAPE 80 group
Neuron numbers	**1241.7** **±** **62.4**	**1402.5** **±** **31.4**^*∗*^	**1796.7** **±** **75.1**^#†^

## Data Availability

The datasets used and/or analyzed during the current study are available from the corresponding author on reasonable request.
